# Treatment Efficacy of Dihydroartemisinin–Piperaquine for Uncomplicated *Plasmodium falciparum* and *Plasmodium vivax* Malaria in Timika, Papua, Indonesia

**DOI:** 10.4269/ajtmh.25-0291

**Published:** 2025-11-11

**Authors:** Noy Norman Kambuaya, Muhammad Syawal Satria Ramli, Freis Candrawati, Enny Kenangalem, Pak Prayoga, Agatha Mia Puspitasari, Rintis Noviyanti, Leily Trianty, Ratni Indrawanti, Minerva Simatupang, Reynold R. Ubra, Jenny Hill, Firdaus Hafidz, Jeanne Rini Poespoprodjo

**Affiliations:** ^1^ Timika Research Facility, Papuan Health and Community Development Foundation, Timika, Indonesia;; ^2^ Exeins Health Initiative, Jakarta, Indonesia;; ^3^ Eijkman Research Center for Molecular Biology-BRIN, Jakarta, Indonesia;; ^4^ Department of Child Health, Faculty of Medicine, Public Health and Nursing, Universitas Gadjah Mada/Dr. Sardjito Hospital, Yogyakarta, Indonesia;; ^5^ Indonesian Ministry of Health, Jakarta, Indonesia;; ^6^ Mimika District Health Office, Timika, Indonesia;; ^7^ Department of Clinical Sciences, Liverpool School of Tropical Medicine, Liverpool, United Kingdom;; ^8^ Department of Health Policy and Management, Faculty of Medicine, Public Health and Nursing, Universitas Gadjah Mada, Yogyakarta, Indonesia;; ^9^ Centre for Child Health-PRO, Faculty of Medicine, Public Health and Nursing, Universitas Gadjah Mada, Yogyakarta, Indonesia;; ^10^ Mimika District Hospital, Timika, Indonesia

## Abstract

Dihydroartemisinin–piperaquine (DP), the first-line treatment for uncomplicated malaria in Timika, Papua, Indonesia, has also been used for intermittent preventive treatment in pregnancy (IPTp-DP) since February 2022. Concerns about the potential emergence of drug resistance associated with this dual policy prompted the present study, which was conducted to assess DP efficacy in treating uncomplicated *Plasmodium falciparum* (*P. falciparum*) and *Plasmodium vivax* (*P. vivax*) malaria in the general population 15 months after IPTp-DP was introduced. Between May and December 2023, the current study recruited 75 *P. falciparum* and 75 *P. vivax* malaria patients, who received supervised DP treatment for 3 days. Clinical and laboratory data were collected daily (on days 1, 2, 3, and 7) and then weekly for 6 weeks. Molecular analysis was performed to detect genetic markers of *P. falciparum* resistance to DP and distinguish between recrudescence and reinfection. A total of 68 *P. falciparum* and 58 *P. vivax* patients completed their day 42 follow-up. The cumulative risk of same-species recurrence by day 42 was 1.5% (95% CI: 0–7.9%) in *P. falciparum* patients (polymerase chain reaction-adjusted) and 5.2% (95% CI: 1.1–14.1%) for *P. vivax* patients (unadjusted). No patients exhibited parasitemia on day 3. No *P. falciparum* isolates carried *kelch 13* gene mutations or exhibited increased *plasmepsin* 2–3 copy numbers on either day 0 (0/75) or at recurrence (0/2). At the current level of IPTp-DP coverage (824 doses administered), there was no evidence of high treatment failure rates or the selection of resistant parasites in patients with uncomplicated malaria treated with DP. Continuous monitoring of DP efficacy remains crucial for both treatment and chemoprevention.

## INTRODUCTION

Malaria is a major public health problem in Timika, Papua Province, Indonesia, including in pregnant women. The annual parasite incidence (API) in 2022 was 440/1,000 population at risk, with *Plasmodium falciparum* (*P. falciparum*) and *Plasmodium vivax* (*P. vivax*) infections being similarly prevalent.^1^ The prevalence of malaria parasitemia at delivery in the area is high (16.8%), and infection is associated with adverse maternal and pregnancy outcomes.^2^ Sulfadoxine–pyrimethamine-resistant strains of *P. falciparum* and chloroquine-resistant strains of *P. vivax* are highly prevalent in this setting.^3^

Dihydroartemisinin–piperaquine (DP) has been the first-line treatment for uncomplicated malaria in Timika since March 2006, and across Indonesia since 2010. Even after 9 years of extensive use, DP remains highly effective against both *P. falciparum* and *P. vivax* infections, with 98% of patients remaining aparasitemic by day 42. No evidence of artemisinin or piperaquine-resistant *P. falciparum* parasites was found in a previous therapeutic efficacy monitoring study.^4^ Beyond its use for treatment, the long half-life of piperaquine makes DP an ideal candidate for preventive therapy in pregnant women and infants in malaria-endemic areas.^5^ A clinical trial in Papua Province conducted between 2013 and 2016 revealed that intermittent preventive treatment in pregnancy with DP (IPTp-DP) reduced the risk of malaria in pregnancy by 77% compared with the National Program’s single screening and treatment strategy.^6^ Given the significant detrimental impact of maternal malaria in this area,^2^ as well as trial evidence supporting IPTp-DP as the most effective strategy to reduce the risk of malaria in pregnancy, 10 community health centers (CHCs) have implemented IPTp-DP as part of their routine malaria prevention in pregnancy program.^7^^,^^8^ Intermittent preventive treatment in pregnancy with DP is administered monthly, starting in the second trimester until delivery.^7^ However, the use of a first-line antimalarial drug for preventive treatment raises concerns about the risk of promoting the de novo selection of new resistance mutations or facilitating the spread of existing drug-resistant parasites within the general population.^9^^–^^11^ Continuous monitoring of DP efficacy and resistance markers is essential to mitigate these risks.

Currently, there is limited evidence regarding the selection of resistant parasites after the use of DP for both treatment and chemoprevention.^9^^,^^12^ In addition, the efficacy of DP treatment has not been evaluated in this setting since 2015.^4^ The aim for the present study was to assess the efficacy of DP treatment of uncomplicated *P. falciparum* and *P. vivax* malaria in a non-pregnant population, 16 years after its introduction and 15 months after the implementation of IPTp-DP.

## MATERIALS AND METHODS

### Study site.

The current study was conducted in Timika, Papua Province, Indonesia, an area with a population of ∼300,000.^13^ The climate in Timika is predominantly rainy throughout the year, and the area is largely forested, encompassing both coastal and mountainous areas. In 2022, the API was 440 per 1,000 population at risk, with an annual total of 132,547 malaria cases. The prevalence of *P. falciparum* and *P. vivax* infections is roughly equal (50:50).^1^

The present study was part of an IPTp-DP implementation study conducted across 10 CHCs in Timika, including Wania CHC.^7^ Wania CHC served as the study site for the clinical trial to evaluate DP treatment efficacy. Malaria is highly endemic in Wania, with the API consistently ranging between 200 and 400 per 1,000 population at risk.^1^ The total population of this subarea was 14,706.^14^ Intermittent preventive treatment in pregnancy with DP has been implemented in Wania CHC since February 2022. Between February 2022 and November 2023, a total of 5,833 antenatal care visits were recorded. During this period, 824 IPTp-DP doses were administered to pregnant women, with 459 women receiving at least one IPTp-DP course.^7^

### Study design.

In the present prospective surveillance study, clinical and parasitological responses to directly observed treatment with DP were evaluated for uncomplicated symptomatic *P. falciparum* and *P. vivax* malaria. The study was designed in accordance with the WHO’s guidelines for efficacy monitoring as part of a routine surveillance program to detect early signs of drug resistance.^15^

Previous efficacy data from 2015 revealed DP treatment failure rates by day 42 of 4.7% for *P. falciparum* and 2% for *P. vivax.*^4^ Given the potential decline in efficacy after 7 years of deployment, a treatment failure rate of 20% was used to calculate the required sample size. Assuming a 95% CI and a precision of ±10%, a minimum of 62 patients per study arm (*P. falciparum* and *P. vivax*) was required. To account for potential loss to follow-up and withdrawals during the 42-day follow-up period, the sample size was increased by 20%, resulting in a target enrollment of a minimum of 74 patients per arm.

### Patients.

Patients aged 1–65 years who weighed more than 5 kg and presented with fever (axillary temperature ≥37.5°C) or a history of fever in the past 24 hours were eligible for enrollment if they had a slide-confirmed parasitemia of >1,000/*µ*L asexual parasites for *P. falciparum* mono-infection and >250/*µ*L asexual parasites for *P. vivax* mono-infection. The exclusion criteria included pregnant and lactating women, patients with severe malaria, and those with comorbidities or severe malnutrition.^15^ For children aged ≤12 years, informed consent was obtained from a parental or legal guardian. For children aged 13 to <18 years, assent was obtained from the child, and informed consent was obtained from their parent or legal guardian. Patients aged *≥*18 years provided their own consent. For illiterate patients, consent was obtained in the presence of a literate witness. All informed consent was provided in the Indonesian language.

### Study procedures.

At enrollment, patient demographic data, symptoms, clinical examination findings, and previous antimalarial medication were documented using a standardized form. Axillary temperature was measured using a digital thermometer. Capillary blood samples were collected via finger prick for malaria microscopy, hemoglobin measurement, and parasite genotyping. Hemoglobin levels were assessed using a portable photometer (HemoCue™ Hb201+ [HemoCue, Brea, CA]) at enrollment and on day 14. For patients with *P. vivax* malaria, glucose-6-phosphate dehydrogenase deficiency status was determined at enrollment using the fluorescent spot test method.^16^

During the first 3 days of supervised DP treatment, patients were examined daily, followed by weekly assessments for 6 weeks. At each visit, a clinical review was conducted, and malaria smears were obtained. Patients were encouraged to return to the clinic at any time if they felt unwell. Microscopic examination of Giemsa-stained blood films was used to quantify asexual parasites per 200 white blood cells (WBCs), with parasitemia calculated based on an assumed WBC count of 7,300/*µ*L. Blood slides were considered negative after 400 high-power fields were examined. A thin smear was also assessed to confirm the parasite species. All slides were read independently by two expert microscopists. Discordant microscopy reading results detected using the Obare methods^17^^,^^18^ would be read by a third microscopist to establish a consensus.

At enrollment and on the day of treatment failure or recurrence, a total of 500 *µ*L of capillary blood samples were collected via finger prick into ethylenediamine tetraacetic acid-coated Microtainers™ (Becton, Dickinson and Company, Franklin Lakes, NJ) for molecular analysis, including speciation, genotyping, and the detection of molecular markers of antimalarial resistance. The polymerase chain reaction (PCR) speciation technique used has been described elsewhere.^4^^,^^19^^,^^20^ To differentiate between recrudescence (same parasite strain) and a newly acquired infection (different parasite strain), *P. falciparum* genotyping was performed using *msp2* and *glurp* markers with standard gel methods, as previously described.^21^^–^^23^ The genotypic profiles of pre- and post-treatment strains were then compared according to the allelic classification, as published elsewhere.^22^

For *P. vivax* cases, a novel genotyping method based on the sequencing of 93 microhaplotypes was performed according to established procedures.^24^^,^^25^ The amplicon library was sequenced using Illumina MiSeq (Illumina, Inc., San Diego, CA), generating 150-base pair (bp) paired-end reads. Using cutadapt (National Bioinformatics Infrastructure Sweden, Stockholm, Sweden),^26^ the reads were filtered to remove adapters and primers, and low-quality bases from the 3′ end of reads were trimmed with a quality cutoff of 10. After filtering, reads with less than 115 bp were removed. The quality-controlled reads were then processed to generate an analysis-ready dataset using Broad’s malaria amplicon familial-relatedness of *P. vivax* parasites was assessed by comparing pre-treatment and post-recurrence samples using identity-by-descent (IBD). Dcifer was used to estimate pairwise IBD for all day 0 and recurrence samples, with day 0 samples used to estimate population allele frequency.^27^ Polymorphisms in the propeller domain of the *P. falciparum kelch* (*K13*) gene, a marker of artemisinin resistance, were identified using Sanger sequencing according to the WorldWide Antimalarial Resistance Network protocol.^28^^–^^31^ Additionally, the copy number variants of *plasmepsin* 2–3 cluster gene, which are associated with piperaquine resistance, were analyzed^32^^–^^37^ using an established protocol.^32^^,^^33^ All procedures were conducted at the Exeins Health Initiative in Jakarta.

### Treatment.

As per national guidelines, DP, containing 40 mg dihydroartemisinin and 320 mg piperaquine (DHP Frimal^®^ [Holley-Cotec, China]), was administered once daily for 3 days. The dosage was weight-based, targeting 2.25 mg/kg of dihydroartemisinin and 18 mg/kg of piperaquine.^38^ All DP doses were administered with biscuits and directly supervised, with patients observed for 30 minutes for signs of vomiting. If vomiting occurred, the dose was readministered. If vomiting recurred, the patient was withdrawn from the study and hospitalized for intravenous artesunate therapy. Patients with *P. vivax* malaria were given unsupervised primaquine treatment at 0.5 mg/kg/day for 14 days, starting on day 28 of the study follow-up period. Patients who experienced therapeutic failure within 28 days of initial treatment were managed according to local guidelines, receiving a 7-day oral quinine regimen (10 mg/kg per dose, three times daily) combined with either doxycycline (2 mg/kg per day, divided into two doses) for individuals over 8 years of age, or clindamycin (5 mg/kg per dose, 3 times daily) for children under 8 years of age. Patients with treatment failure that occurred after 28 days were retreated with DP.

### Endpoints.

The primary endpoints were the PCR-adjusted risk of *P. falciparum* recurrence and the unadjusted risk of *P. vivax* recurrence. Secondary endpoints included the proportion of patients who remained parasitemic on days 1, 2, and 3 post-treatment, gametocyte carriage, hematological recovery, and the proportion of patients with *P. falciparum* carrying *K*13 gene mutations and *plasmepsin* 2–3 copy number variants.

## STATISTICAL ANALYSES

Data were double-entered using EpiData 3.02 software (EpiData Association, Odense, Denmark) and analyzed using SPSS version 25 (IBM^®^, Armonk, NY). Continuous variables were reported as mean (SD) if they were normally distributed and as median (range) if not. Categorical variables were summarized as percentages with 95% CIs. The Kaplan–Meier method was used to estimate the cumulative risk of treatment failure, along with the corresponding 95% CIs. Cases lost to follow-up were censored on their final day of follow-up and were not classified as treatment failures. Early treatment failures and homologous recrudescence, as determined using standard gel methods, were categorized as treatment failures.

## RESULTS

**Figure 1.**
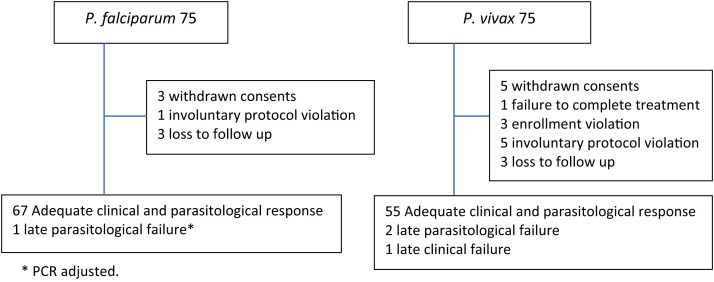
Study profile.

Between May 30, 2023 and December 13, 2023, a total of 1,075 patients with malaria were screened for enrollment, of whom 370 met the eligibility criteria. Informed consent was obtained from 75 patients with uncomplicated *P. falciparum* and 75 with *P. vivax* malaria. After enrollment, eight patients withdrew consent, citing personal reasons, three cases were classified as enrollment violations, six cases were considered involuntary protocol violations, and six patients were lost to follow-up (Figure 1). In the *P. vivax* group, cross-checks of slide readings revealed that one patient had *Plasmodium ovale* (*P. ovale*), one patient had mixed *P. falciparum* and *P. vivax* infections, and one patient had low parasitemia (<250/*µ*L), and these patients were classified as enrollment violations. Involuntary protocol violations in the *P. vivax* group included five patients who were initially excluded because of microscopy results at enrollment (four mixed infections and one *P. ovale* infection) but were later confirmed as *P. vivax* mono-infections via PCR testing. In the *P. falciparum* group, one patient self-administered DP on day 14 without medical advice. After exclusions, the final cohort for analysis included 68 patients with *P. falciparum* malaria and 58 patients with *P. vivax* malaria. The baseline characteristics are presented in Table 1.

**Table 1:** Baseline characteristics of patients at the 42-day follow-up

Baseline Characteristic	*P. falciparum* (*n* = 68)	*P. vivax* (*n* = 58)
Male patients	39 (57.4)	33 (56.9)
Age (years), median (range)	17.5 (3–48)	18.5 (1–49)
<5 years	1 (1.5)	8 (13,6)
5-<15 years	29 (42.6)	17 (29.3)
≥15 years	38 (55.9)	33 (56.9)
Axillary temperature (°C), median (range)	37.1 (36.5–40)	36.7 (36.5–40)
History of fever in the last 24 hours	68 (100)	58 (100)
Parasitemia per *µ*L of blood, mean (95% CI)	54,994 (39,237–70,752)	11,129 (6,251–16,107)
Geometric mean parasitemia per *µ*L of blood (95% CI)	10,196 (9,879–10,513)	8,618 (8,310–8,926)
Hb (g/dL), mean (SD)	12.7 (2.7)	12.3 (2.7)
Gametocyte carriage	7 (10.3)	51 (87.9)

*P. falciparum* = *Plasmodium falciparum*; *P. vivax* = *Plasmodium vivax*. Data are presented as numbers (%) unless otherwise indicated.

### Treatment efficacy.

Overall, the PCR-adjusted cumulative risk of treatment failure (classified as recrudescence by genotyping) with the same species by day 42 in 68 patients with *P. falciparum* malaria was 1.5% (95% CI: 0–7.9%; Table 2). Among 68 patients with *P. falciparum* malaria, 11 experienced recurrences between day 28 and day 42, including three with *P. falciparum*, six with *P. vivax*, one with mixed *P. falciparum* and *P. vivax* infections, and one with *Plasmodium malariae* (*P. malariae*). After PCR adjustment, the number of recurrences declined to eight cases, including two with *P. falciparum* (one reinfection and one recrudescence; Supplemental Table 1) and six with *P. vivax*. One *P. falciparum* recurrence on day 35 was confirmed to be the same strain as that identified at enrollment and was classified as late treatment failure (Supplemental Table 1). None of the *P. falciparum* recurrences occurred within the first 28 days of treatment. The risk of *P. vivax* recurrence after contracting *P. falciparum* malaria by day 42 was 8% (6/68 patients).

**Table 2:** Cumulative risk of treatment failure according to initial species of infection

Type of Treatment Failure	*P. falciparum*	*P. vivax*
Early treatment failure	0	0
Late parasitological failure	1.5% (1/68; 0–7.9%)	3.4% (2/58; 0.4–11.9%)
Late clinical failure	0	1.7% (1/58; 0–9.2%)
Late treatment failure by day 28	0	5.2% (3/58; 1.1–14.4%)
Late treatment failure by day 42	4.4% (3/68; 1.0–12.4%)	5.2% (3/58; 1.1–14.4%)
Late treatment failure by day 42, PCR-adjusted	1.5% (1/68; 0–7.9%)	Not applicable

PCR = polymerase chain reaction; *P. falciparum* = *Plasmodium falciparum*; *P. vivax* = *Plasmodium vivax*.

The unadjusted cumulative risk of recurrence with the same species by day 42 in 58 patients with *P. vivax* malaria was 5.2% (95% CI: 1.1–14.1%; Table 2). Among 58 patients with *P. vivax* malaria, fewer recurrences were observed compared with those with *P. falciparum* cases. By day 28, four recurrences were recorded, three patients had confirmed *P. vivax* recurrences (two patients were confirmed as *P. vivax* by PCR, one patient had no blood sample available for molecular analysis, and one patient had *P. malariae* [PCR test result was negative]). Novel genotyping methods for *P. vivax* used in the present study revealed that in the two *P. vivax* recurrences with available samples, one was classified as a possible relapse (day 28), and one was classified as a possible reinfection (day 21; Supplemental Table 2). Because PCR genotyping could only be performed on two of the three *P. vivax* recurrences, the PCR-adjusted treatment efficacy for *P. vivax* malaria could not be quantified. The median total dose of dihydroartemisinin and piperaquine received by all patients was 7.3 mg/kg (range: 5.8–10.8 mg/kg) and 58.1 mg/kg (range: 46.6–86.8 mg/kg), respectively.

For *P. falciparum* malaria, 19.1% (13/68) of patients were aparasitemic after 24 hours, rising to 85.3% (58/68) by 48 hours. Parasite clearance rates were higher in patients with *P. vivax* malaria than those with *P. falciparum* infections: 58.6% (34/58) of patients were aparasitemic after 24 hours, and 94.8% (55/58) by 48 hours. By day 3, all patients had become aparasitemic.

### Gametocyte carriage.

**Figure 2.**
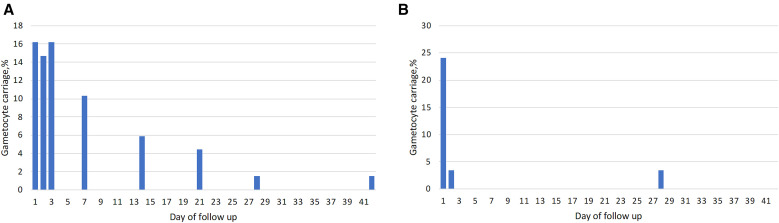
Proportion with gametocyte carriage in patients with *P. falciparum* malaria (left) and *P. vivax* malaria (right).

Gametocyte carriage at enrollment was higher in patients with *P. vivax* malaria (87.9%; 51/58) than in those with *P. falciparum* malaria (10.3%; 7/68). After treatment, gametocyte carriage in patients with *P. falciparum* malaria increased to 16.2% (11/68), 14.7% (10/68), and 16.2% (11/68) on days 1, 2, and 3, respectively. The proportion of patients with gametocytemia declined to 10.3% (7/66) on day 7 and continued to decline to 5.9% (4/67), 4.4% (3/64), 1.5% (1/66), and 1.5% (1/65) on days 14, 21, 28, and 42, respectively (Figure 2). All but one patient received single-dose primaquine for gametocyte clearance on day 28 (67/68). By contrast, gametocyte carriage in *P. vivax* declined rapidly after treatment, to 24.1% (14/58) and 3.4% (2/58) on days 1 and 2, respectively, with two patients still having gametocytes on day 28 (2/57; 3.4%). A 6-year-old girl with *P. falciparum* malaria had persistent gametocytemia until day 28 and a *P. falciparum* recurrence with a different strain on day 42, suggesting likely reinfection.

### Hematological recovery after treatment.

Hemoglobin concentration data at enrollment and on day 14 were available for 65 patients with *P. falciparum* malaria and 55 patients with *P. vivax* malaria. The mean hemoglobin concentration at enrollment was 12.7 g/dL (SD 2.7) in patients with *P. falciparum* malaria and 12.3 g/dL (SD 2.7) in those with *P. vivax* malaria. The mean reduction in hemoglobin concentration on day 14 was 1.3 g/dL (SD 1.9) in *P. falciparum* patients and 0.6 g/dL (SD 1.8) in *P. vivax* patients. Three patients with *P. falciparum* malaria and one with *P. vivax* malaria had hemoglobin reductions of ≥5 g/dL, all of whom had high hemoglobin concentrations at enrollment and none of whom had hemoglobin concentrations below 7 g/dL on day 14 (Table 3).

**Table 3:** Patient with hemoglobin drops ≥5 g/dL

Code	Species	Hb on Day 0 (g/dL)	Hb on Day 14 (g/dL)	Hb Drops (g/dL)
TES 054	*P. falciparum*	16.7	10.7	6
TES 061	*P. falciparum*	19.9	13.5	6.4
TES 071	*P. falciparum*	14.4	7.9	6.5
TES 117	*P. vivax*	16.3	10.3	6

Hb = hemoglobin; *P. falciparum* = *Plasmodium falciparum*; *P. vivax* = *Plasmodium vivax*; TES = therapeutic efficacy study.

### Adverse events.

Two cases of vomiting occurred after the first DP dose in a 2-year-old child and a 6-year-old child. In both cases, DP was successfully readministered. No severe adverse events were reported.

### Molecular marker of resistance.

No *K13* gene mutations in the propeller domains of *P. falciparum* or increased copy numbers of *plasmepsin 2–3* genes were detected in *P. falciparum* samples either on day 0 (0/75) or at recurrence (0/2).

## DISCUSSION

The present study revealed that nearly two decades after its introduction for treatment and 15 months after its small-scale introduction for IPTp, DP retained its efficacy for treating both *P. falciparum* and *P. vivax* malaria in Timika (Papua, Indonesia), with a day 42 treatment failure rate below 5%. In addition, there was no evidence of artemisinin derivative- or piperaquine-resistant *P. falciparum* parasites circulating in the study area.

None of the *P. falciparum* and *P. vivax* malaria patients remained parasitaemic on day 3, indicating no delay in parasite clearance associated with artemisinin resistance.^39^^,^^40^ However, the day 2 positivity rate among patients with *P. falciparum* malaria was higher in the present study (14.7%) compared with 2% reported in the 2015 study, although no such increase was observed in *P. vivax*.^4^ This delayed parasite clearance was not likely due to the level of parasitemia because the geometric mean parasitemia across the two efficacy studies was similar.

Gametocyte carriage at enrollment was much higher than in the 2015 study for both *P. falciparum* (10.3% versus 3.3%) and *P. vivax* (87.9% versus 26.5%) malaria patients.^4^ This could be attributed to previous ineffective DP treatment, possibly due to poor adherence in unsupervised settings.^41^ Some study authors have recommended that some patients discontinue DP after the first or second dose once their clinical symptoms improve, leading to recurrent malaria.^41^^,^^42^ Another possible explanation for the higher gametocyte carriage at enrollment could be delayed clinical presentation due to partial clinical immunity, which influences health-seeking behavior.^43^^,^^44^^,^^45^ In areas with high malaria transmission, individuals may develop a degree of immunity that delays symptom onset, leading them to seek treatment later in the course of infection. The study population primarily lived in peri-urban areas, where malaria transmission is typically higher (API >200/1,000 population) compared with the urban setting (API 50–100/1,000 population) of the previous therapeutic efficacy study.^46^ Asymptomatic carriage and the gradual development of clinical immunity from repeated malaria exposure are well-documented in high transmission areas.^43^^–^^45^ This could contribute to delayed treatment and, consequently, higher gametocyte carriage at the time of presentation. With the current level of efficacy (failure rate <10%), resistance development might be slowed by ensuring that patients receive the correct dose and adhere to the full course of treatment.^47^^,^^48^ However, adding an alternative artemisinin-based combination therapy (ACT) option to the national guideline would be beneficial if resistance emerges. The most recently approved ACT, pyronaridine–artesunate, exhibited good efficacy in areas with documented resistance to other ACTs.^48^^–^^50^

Although all asexual parasitemia was cleared on day 3 after treatment, *P. falciparum* gametocyte clearance appeared to be delayed compared with the 2015 study. In the present study, gametocytes were detected at multiple time points, including on days 1, 2, 3, 7, 14, 21, 28, and 42, with the highest prevalence of 16.2% on days 1 and 3. In contrast, the authors of the 2015 study reported gametocytes only on days 1, 2, 7, and 14, with a peak prevalence of 12.3% on day 2.^4^ Furthermore, gametocytes persisted until day 42 in one patient, whereas in 2015, the longest persistence lasted until day 14 (one patient). The delay could be due to the slower rate of *P. falciparum* parasite clearance after 24 hours after treatment in the present study (80.9% with parasitemia) compared with the 2015 efficacy study (37.3%).^4^ In both studies, single-dose primaquine treatment of patients with *P. falciparum* malaria was administered on day 28. A higher proportion of patients with *P. vivax* malaria had gametocytes on day 1 (24.1%) compared with only 6% in the 2015 study.^4^ However, this proportion dropped to 3.4% by day 2. In the current study, gametocytes were detected on days 2 and 28, whereas in 2015, they were detected on days 2, 7, and 14.^4^ The higher gametocytemia at enrollment in the current study likely contributed to the higher and persistent gametocyte carriage observed.^51^ The possibility of resistant gametocytes is unlikely because no genetic markers associated with artemisinin derivatives or piperaquine resistance were detected in *P. falciparum* parasites in the present study. This suggests that the differences in gametocyte persistence may be influenced by baseline gametocytemia and parasite clearance rates, rather than drug resistance.

In the current study, *P. vivax* recurrences occurred after day 21, coinciding with the waning post-treatment prophylactic effect of piperaquine.^52^ The relatively high rate of *P. vivax* recurrences following *P. falciparum* infections (8.8%) suggests a high rate of hypnozoite carriage in this area, likely due to an ineffective radical cure.^53^^,^^54^ *Plasmodium vivax* malaria remains challenging to control because of its ability to relapse via activation of dormant hypnozoites in liver hepatocytes without new infections.^53^ The only available treatment to clear hypnozoites in this region is primaquine for 14 days, but its effectiveness is compromised by poor treatment adherence^55^ and suboptimal dosing (3.5 mg/kg/day) in this setting.^56^^,^^57^

Evidence from a trial in Uganda suggests that IPTp-DP may contribute to the selection of drug-resistant parasites. The prevalence of *P. falciparum* genetic polymorphism linked to reduced piperaquine sensitivity (*pfmdr1* N86Y, *pfmdr1* Y184F, and *pfcrt* K76T) significantly increased compared with pre-IPTp-DP levels.^58^ Interestingly, these same genetic mutations are also linked to reduced sensitivity to amodiaquine, an aminoquinoline like piperaquine, which has been widely used for malaria treatment in most of Africa in artesunate–amodiaquine combination therapy.^10^^,^^11^^,^^52^ This suggests that in African settings, IPTp-DP may accelerate the selection of preexisting resistant parasites associated with aminoquinoline rather than driving the emergence of new mutations.^10^ On the other hand, IPT-DP is not part of routine malaria control strategies in endemic areas outside Africa; therefore, no data are available on its impact on DP resistance.^6^^,^^48^

The emergence of drug resistance is more likely due to inappropriate drug use for treatment (i.e., case management), where a large number of parasites are exposed to subtherapeutic drug levels, rather than in preventive strategies like IPTp-DP.^47^ In this setting, although DP has been widely used as the first-line treatment in the general population for nearly two decades, there is no evidence of circulating drug-resistant *P. falciparum* or delayed parasite clearance. This suggests that introducing DP as IPTp in asymptomatic pregnant women is unlikely to drive de novo resistant mutations, given that parasitemia levels in this population are typically lower.^5^^,^^10^^–^^12^^,^^47^^,^^59^

In the present study, DP treatment efficacy is evaluated in the general population; therefore, a direct link between IPTp-DP and the risk of drug resistance cannot be established. However, given that no resistant *P. falciparum* parasites were previously detected in this area,^4^ and considering that resistance would likely emerge from treatment rather than prevention,^47^ treatment efficacy is considered to be more relevant for emerging resistance. Although IPTp-DP could theoretically generate new resistant parasites, the likelihood of these less-fit mutant parasites transmitting resistant gametocytes to the general population and establishing infections is probably very low.^59^^,^^47^ The relatively low IPTp-DP coverage achieved during the implementation pilot suggests a minimal risk of driving drug resistance, limiting the interpretation of its possible impact on resistance development.

The study findings may not be generalizable to other settings. Dihydroartemisinin–piperaquine has also been widely used in other parts of Indonesia; therefore, additional efficacy data from other settings would provide a more comprehensive picture of overall DP efficacy. Ideally, therapeutic efficacy studies should be conducted every 24 months to enable the timely evaluation of antimalarial efficacy, as recommended by the WHO.^15^ However, these studies are resource-intensive and technically challenging, which limits the availability of efficacy data.

## CONCLUSION

The present study revealed that DP remained efficacious after almost 20 years of use as a malaria treatment in this setting. At the current level of coverage, the possible role of IPTp-DP in driving the emergence of drug-resistant parasites could not be determined. Therefore, continuous periodic monitoring of treatment and chemoprevention efficacy, as well as molecular surveillance of resistant parasites, remains essential in areas where the same antimalarial drug is used for both treatment and prevention.

## Supplemental Materials

10.4269/ajtmh.25-0291 Supplemental Materials
