# Supplementary material for: Treatment Efficacy of Dihydroartemisinin–Piperaquine for Uncomplicated Plasmodium falciparum and Plasmodium vivax Malaria in Timika, Papua, Indonesia
Source: Am J Trop Med Hyg. 2025 Nov 11;114(1):115–22. doi: 10.4269/ajtmh.25-0291 (PMC12781433; doi:10.4269/ajtmh.25-0291)
Supplement: Supplemental Materials [file tpmd250291.SD1.pdf]

Supplementary Table 1. Summary of *P. falciparum* genotyping result for all of the pairing samples

Sample Code	Day	Allele Code		Genotyping Result	Clinical Outcome
		glurp*	***msp2***$^{+}$		
TES-051	0	9	6 and 4	3D7-like strain & FC27-like strain	Recrudescence
	35 – Recurrence	9	6	3D7-like strain	
TES-066	0	5	1	FC27-like strain	Reinfection
	42 – Recurrence	9	6	3D7-like strain	

* Size ranges of the Allele Code (*glurp*) 5 = 640 bp – 699 bp; 9 = 880 bp – 939 bp;

$^{+}$ Size ranges of the Allele Code (*msp2*) 1 = 400 bp – 439 bp; 4 = 520 bp – 559 bp; 6 = 600 bp – 639 bp

Supplementary Table 2. Summary of *P. vivax* genotyping result by microhaplotype markers

Sample Code	Day	IBD Shared	Microhaplotype Alleles Observed	Clinical Outcome
TES-091	0	96.8%	Refer to Supplementary File 2.	Possibly a relapse case
	28 – Recurrence			
TES-109	0	0.4%	Refer to Supplementary File 3.	Possibly a reinfection case
	21 - Recurrence			
